# Cardiac and Nephrological Complications Related to the Use of Antiangiogenic and Anti-Programmed Cell Death Protein 1 Receptor/Programmed Cell Death Protein 1 Ligand Therapy

**DOI:** 10.3390/genes15020177

**Published:** 2024-01-28

**Authors:** Paulina Stachyra-Strawa, Lidia Szatkowska-Sieczek, Paweł Cisek, Paweł Gołębiowski, Ludmiła Grzybowska-Szatkowska

**Affiliations:** 1 Department of Radiotherapy, Medical University of Lublin, Chodźki 7, 20-093 Lublin, Poland; paulina.stachyra@gmail.com (P.S.-S.); pcisek@interia.eu (P.C.); golebiowski.pawel@wp.pl (P.G.); 2 Clinical Department of Cardiology, 4th Military Hospital, Rudolfa Weigla 5, 50-981 Wroclaw, Poland; lidia.szatkowska@op.pl

**Keywords:** anti-VEGF therapy, cardiotoxicity, TKI, ICI, neurotoxicity

## Abstract

The ability to undergo neoangiogenesis is a common feature with all cancers. Signaling related to vascular endothelial growth factors (VEGF) and their receptors (VEGFR) plays a key role in the process of tumor neoangiogenesis. A close relationship has been demonstrated between excessive VEGF levels and the induction of immunosuppression in the tumor microenvironment. The use of drugs blocking the VEGF function, apart from the anticancer effect, also result in adverse effects, in particular related to the circulatory system and kidneys. Cardiac toxicity associated with the use of such therapy manifests itself mainly in the form of hypertension, thromboembolic episodes and ischemic heart disease. In the case of renal complications, the most common symptoms include renal arterial hypertension, proteinuria and microangiopathy. Although these complications are reversible in 60–80% of cases after cessation of VSP (VEGF pathway inhibitor) therapy, in some cases they can lead to irreversible changes in renal function, whereas cardiac complications may be fatal. Also, the use of PD-1/PD-L1 inhibitors may result in kidney and heart damage. In the case of cardiac complications, the most common symptoms include myocarditis, pericarditis, arrhythmia, acute coronary syndrome and vasculitis, while kidney damage most often manifests as acute kidney injury (AKI), nephrotic syndrome, pyuria or hematuria. The decision whether to resume treatment after the occurrence of cardiovascular and renal complications remains a problem.

## 1. Introduction

The ability to undergo neoangiogenesis is a common feature to all cancers. The tumor’s blood vessel network shows structural, functional and biochemical abnormalities. The vessels have a tortuous course, dead-ending branches, a reduced number of pericytes and smooth muscles and large fenestrations of endothelial cells. These disorders determine vascular leakage and unstable blood flow, which, in turn, leads to tissue hypoxia, development of acidosis and increased interstitial pressure in the tumor microenvironment (TME) [1,2]. Such conditions may reduce the effectiveness of the therapy, including immunotherapy and antiangiogenic treatment, so its normalization may determine the success of the therapy. Malfunctioning vessels and impaired perfusion may limit the penetration of cytotoxic drugs and immune cells into the tumor, which limits their anticancer activity [3]. Therefore, it seems important to search for and use drugs with anti-angiogenic effects in oncological therapy. A pivotal role in the process of tumor neoangiogenesis is played by signaling related to vascular endothelial growth factors (VEGF) and their receptors (vascular endothelial growth factor receptors, VEGFR) [4]. A close relationship has been demonstrated between excessive VEGF levels and the induction of immunosuppression in the TME. On the one hand, VEGF promotes angiogenesis, and the abnormal blood vessels formed in this process result in the development of hypoxia and low pH, which has an immunosuppressive effect. On the other hand, angiogenic factors, including VEGF, have the ability to modulate immune cells towards immunosuppression. VEGF creates a pro-tumor environment by inhibiting the activation of cytotoxic T lymphocytes (CTLs) and increasing the number and function of suppressive regulatory lymphocytes (Tregs), tumor-associated macrophages (TAMs) and the number of T cells CD4+ memory. Moreover, these cells also have the ability to release VEGF. The PD1/PD-L1 (programmed cell death protein 1 receptor/programmed cell death protein 1 ligand) pathway is often activated in the TME as a mechanism to evade anti-tumor immune responses. Increased PD-L1 expression is observed on both TAM cells and tumor cells. Therefore, there is a close relationship between abnormal angiogenesis and the immunosuppressive TME [3]. Consequently, it seems rational to combine antiangiogenic therapy with immune checkpoint inhibitors (ICPIs). The first anti-VEGF drug approved for treatment was a humanized monoclonal antibody that binds to VEGF—bevacizumab. It was approved by the Food and Drug Administration (FDA) in 2004 for the treatment of advanced colorectal cancer in combination with chemotherapy [5]. In the following years, drugs with a different mechanism of action were used, including aflibercept—a recombinant fusion protein which acts as a false VEGF-binding receptor, characterized by greater affinity than native receptors (so-called VEGF-TRAP) [6]. Drugs that directly block the VEGFR receptor include the human monoclonal antibody ramucirumab [6]. Another group of drugs are low-molecular-weight tyrosine kinase inhibitors (anti-VEGF multi-target TKIs), which block the binding of ATP molecules by active kinase centers, are characterized by lower specificity and affect many proteins at the same time. These drugs include sunitinib and sorafenib [7]. Among the checkpoint inhibitors, the most commonly used drugs are those that block the action of PD-1: nivolumab and pembrolizumab, and the anti-PD-L1 inhibitor—atezolizumab [8]. Examples of anti-VEGF and anti-PD-1 drugs that are most often used in the authors’ daily practice, their targets, possible toxicity and use in cancer therapy are presented in Table 1.

Given the fact that VEGF is responsible for homeostasis related to blood vessels, the use of drugs blocking its function, in addition to the anticancer effect, will also be reflected in the form of adverse effects, in particular related to the circulatory system and kidneys. Cardiac toxicity related to the use of therapy manifests itself mainly in the form of hypertension, thromboembolic episodes and ischemic heart disease [10]. In the case of renal complications, the most frequently observed are renal arterial hypertension, proteinuria and microangiopathy [11]. Also, the use of PD-1 inhibitors may result in kidney and heart damage. In the case of cardiac complications, the most common symptoms include myocarditis, pericarditis, arrhythmia, acute coronary syndrome and vasculitis [12], while kidney damage most often manifests itself as acute kidney injury (AKI), nephrotic syndrome, pyuria or hematuria [13].

## 2. Mechanism of Angiogenesis in Tumors and Immune Checkpoints

The mechanism of neoangiogenesis is largely dependent on the concentration of VEGF. It can activate cellular pathways that regulate cell division, migration, survival and apoptosis. These pathways include the PI3K/Akt signaling pathway (the phosphatidylinositol 3-kinase and serine-threonine protein kinase Akt pathway) and RAS/MAPK/ERK (rat sarcoma protooncogene/mitogen-activated protein kinase/extracellular signal-regulated kinase) by mutation or constitutive activation of their components [14].

The VEGFR family includes five types of receptors, of which VEGFR-1 and VEGFR-2 play a role in angiogenesis. The binding of VEGF to VEGFR on the tumor blood vessel increases vascular permeability and activates the proliferation and migration of vascular endothelial cells [4]. It has been shown that increased VEGF concentration results in “upregulation” of the VEGFR-2 receptor [15]. The main factor stimulating VEGF is hypoxia. Chronic oxygen deficiency in cells increases the production of hypoxia-inducible factor 1 (HIF-1), which, in turn, is a transcription factor for increasing the production and release of VEGF [16]. The increase in VEGF activation affects the activation of pathways responsible for neoangiogenesis in vascular endothelial cells, which is intended to counteract the effects of hypoxia. This increases the efficiency of endothelial cell proliferation, growth and migration processes and vascular permeability [16]. The VEGF family includes: VEGFA, VEGFB, VEGFC, VEGFD and PlGF (placental growth factor). Signaling by VEGFA is mainly associated with the stimulation of VEGFR-2 [17]. VEGF can be produced by tumor cells, as well as endothelial and stromal cells. Immune system cells also have the ability to produce VEGF [3]. Moreover, cytokines such as VEGF that enter the systemic circulation from the TME may also enhance the extravasation of metastatic tumor cells from blood vessels in distant organs [3]. Circulating VEGF and the soluble form of VEGFR-2 can be used as a predictor of response to antiangiogenic treatment [18].

The RAS/MAPK/ERK pathway is responsible for transmitting signals from the cell surface to its nucleus, controlling the processes of proliferation, differentiation, migration and survival. The combination of a ligand with a receptor tyrosine kinase (RTK) protein results in the phosphorylation of the RAS (rat sarcoma) protein, which in turn activates the RAF (rapidly accelerated fibrosarcoma) protein with serine-threonine kinase activity. The RAF protein has three isoforms: ARAF, BRAF and CRAF, among which BRAF is the strongest activator of MEK (mitogen-activated kinase). The activated RAF kinase phosphorylates MEK1 and MEK2, which then activate the ERK1 and ERK2 kinases (extracellular signal-regulated kinases). Activated ERK kinases transmit a signal to the cell nucleus, which results in increased expression of genes responsible for cell growth and survival [19]. Among all RTKs, a special role of the vascular endothelial growth factor receptor (VEGFR) and its ligands is indicated in the pathogenesis of cancer [20]. The presence of KRAS mutations is expected to increase VEGF activity, thus promoting the process of tumor neoangiogenesis [21].

**Figure 1:**
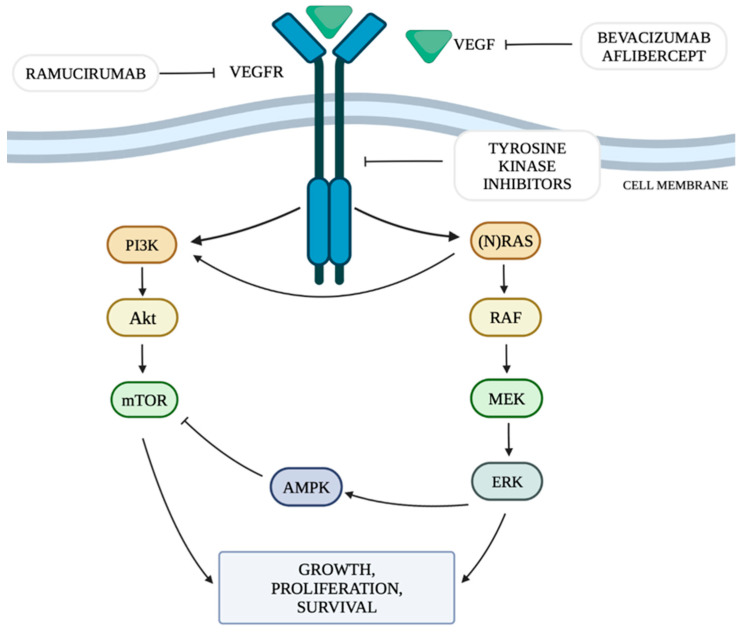
RAS/MAPK/ERK and PI3K/Akt/mTOR pathway and targets for anti-VEGF therapy. Own study based on [6,7,8,19,22].

The phosphatidylinositol 3-kinase and serine-threonine protein kinase Akt (PI3K/Akt) signaling pathway is one of the main metabolic pathways whose disturbances are observed in numerous cancers. They are related to the increased expression of genes whose products are involved in the PI3K/Akt pathway [22]. PI3K is regulated mainly by RTK. The PI3K family includes three classes of kinases, of which class I is the best known and the most important in the context of oncogenesis and pathogenesis of other diseases [23]. In class I PI3K, we can distinguish two subgroups: IA, which includes the α, β and δ isoforms and IB, which includes the γ isoform [23]. PI3Kα plays a key role in cellular pathways related to angiogenesis, growth and metabolism, being the isoform most involved in the process of oncogenesis. Ligands that activate the pathway by attaching to RTKs include VEGF, among others. The activation of PI3K leads to an increase in the production of phosphatidylinositol 3,4,5-trisphosphate (PIP3) by phosphorylation of phosphatidylinositol 4,5-bisphosphate (PIP2). PIP3 is responsible for increasing recruitment to the cell membrane and activation of serine/threonine kinase Akt, also called protein kinase B (PKB). The Akt kinase family includes three proteins: Akt-1, Akt-2 and Akt-3. Activated Akt kinase regulates, among others, the activity of a pro-apoptotic protein located on the outer mitochondrial membrane from the Bcl-2 family—the BAD protein (Bcl-2-associated death promoter)—and the serine-threonine kinase mTOR (serine/threonine kinase mammalian target of rapamycin), thus regulating the processes of apoptosis, angiogenesis and proliferation. The mTOR kinase functions as a catalytic subunit in two different protein complexes, mTORC1 and mTORC2 (mTOR complex 1,2). The PI3K/Akt pathway is controlled by the PTEN phosphatase encoded by the tumor suppressor gene PTEN. PTEN phosphatase is responsible for the conversion of PIP3 to PIP2 by catalyzing the separation of the 3′-phosphate group from PIP3, thus acting as a negative regulator of PI3K. When all components of the pathway function properly, they stop the proliferation process and initiate apoptosis [23]. Figure 1 shows the discussed cellular pathways and the site of action of selected anti-VEGF drugs.

**Figure 2:**
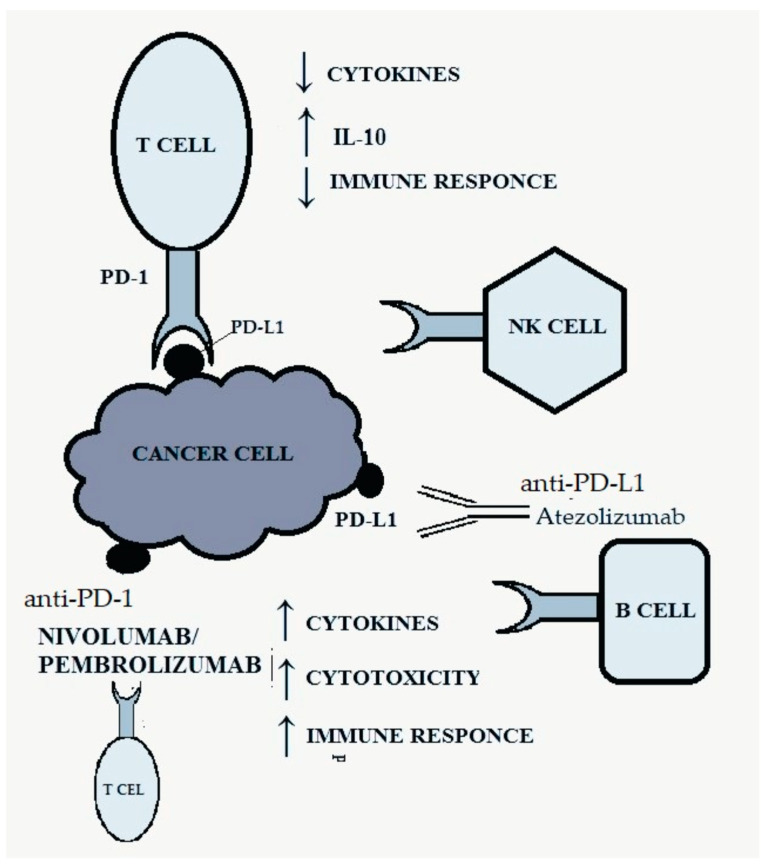
PD-1/PD-L1 pathway and targets for anti-PD-1/PD-L1 therapy. Own study based on [8,24,25].

The PD-1 receptor, belonging to the CD28 family, is present on B lymphocytes, CD4+ and CD8+ T lymphocytes, natural killer (NK) cells, monocytes and activated dendritic cells and is responsible for inhibiting the anti-tumor response [19,24]. It has two ligands: PD-L1 and PD-L2 (programmed death-ligand 1, 2), the first of which is much more widespread and occurs on many different tissues [25]. The combination of the ligand with the PD-1 receptor results in inhibition of the activity of the immune system by reducing the production of cytokines and increasing the synthesis of IL-10, which inhibits the immune response [8,19]. Activation of the PD-1-related pathway allows cancer cells to evade the body’s defense response by negatively regulating effector T cells [8,19]. Figure 2 shows the discussed pathway and the site of action of anti-PD-1/PD-L1 drugs.

## 3. Cardiotoxicity Associated with the Use of Anti-VEGF Therapy

The use of vascular endothelial growth factor inhibitors (VEGFI) and TKIs in oncological therapy may lead to cardiotoxicity expressed by arterial hypertension, left ventricular systolic dysfunction, heart failure and arterial and venous thromboembolism, as well as prolongation of the QTc interval and arrhythmia [26]. The most frequently observed adverse effect associated with the use of VEGFI in relation to the cardiovascular system is hypertension. It is estimated that it affects up to 80–90% of patients treated with such therapy [27]. In a large meta-analysis of 77 clinical trials of VEGF signaling pathway (VSP) inhibitors, severe hypertension occurred in 7.4% of patients, arterial thromboembolism in 1.8%, myocardial ischemia in 1.7% and myocardial failure in 2.3%. The use of VSP inhibitors was associated with a 5.6-fold increase in the risk of severe hypertension, a 2.8-fold increase in the risk of myocardial ischemia, a 1.5-fold increase in the risk of arterial thromboembolism and a 1.4-fold increase in the risk of myocardial failure [28]. The development of hypertension contributes to the dilatation of the ascending aorta, hypertrophy of the interventricular septum and hypertrophy of the left ventricular muscle, which are visible on cardiac ultrasound [29]. Changes typical of this type of myocardial remodeling, characteristic of left ventricular hypertrophy, can be observed on cardiac ultrasound [30]. Studies have shown that EKG is not a reliable way to assess cardiac hypertrophy [31,32]. Echocardiography is still the only reliable assessment of cardiac hypertrophy [31,32].

Meta-analyses of data from randomized clinical trials showed that the use of TKIs, such as sunitinib, sorafenib or pazopanib, was associated with an increased risk of cardiac complications, and due to the lower specificity of the action of TKIs than anti-VEGF antibodies, they may cause even greater damage [10]. The most frequently observed events were hypertension, arterial thrombotic events and decreased left ventricular ejection fraction. Interestingly, there was no increase in the risk of venous thrombotic events associated with their use [10]. In one of the studies available in the literature, 829 patients with renal cell carcinoma treated with TKIs (pazopanib, sunitinib or sorafenib) and 2601 patients treated with bevacizumab were analyzed. During the one-year follow-up, in the group of patients treated with TKIs, 81 cases of major adverse cardiovascular events (MACEs) were recorded (CCI: 9.79%), of which 33 cases were thromboembolic events (CCI: 3.99%), 22 cases cardiac arrhythmia (CCI: 2.66%), 13 pulmonary embolism (CCI: 1.57%) and 13 heart failure (CCI: 1.57%). The incidence of adverse cardiovascular events was higher in the group of patients treated with TKIs than in the group of patients treated with bevacizumab, where a total of 176 events were recorded (CCI: 6.77%), with a predominance of thromboembolic complications (70 cases, CCI: 2.69%). The frequency of MACEs increased with the duration of therapy, both in the group of patients treated with TKIs (4.34% after 3 months, 9.79% after 1 year) and bevacizumab (2.38% after 3 months, 6.77% after 1 year). Interestingly, the incidence of arrhythmias, pulmonary embolism and heart failure associated with TKI use plateaued after 6 months of therapy, while the incidence of thromboembolic events continued to increase. The risk group included patients >65 years of age [33]. During sunitinib treatment, changes in blood pressure were most pronounced during the first three cycles, and the greatest increases in the incidence of grade 3 hypertension and grade 3 cardiac dysfunction were observed in cycles 2 and 3 [10].

The actual incidence of left ventricular systolic dysfunction (LVSD) associated with VEGFI may range from 10 to 20%, and the incidence of heart failure is approximately 1–5% [26].

The incidence of heart failure in the case of bevacizumab was 2–4%, while in the case of TKI use it was 3–8%, and diabetics were identified as a group at particular risk of myocardial blood supply disorders, probably due to progressive myocardial fibrosis and deterioration of heart muscle function associated with diabetes [10]. Interestingly, the use of TKIs may result in a decrease in glucose levels, even leading to the possibility of discontinuing hypoglycemic drugs in diabetic patients [34]. A meta-analysis of 3784 breast cancer patients treated with bevacizumab in randomized, controlled trials found an incidence of “high-grade” congestive heart failure of 1.6% with bevacizumab and 0.4% with placebo. The term “high grade” refers to heart failure grade 3 or higher according to the National Cancer Institute’s (NCI) Common Terminology Criteria for Adverse Events, which includes patients with LVEF < 40% and a spectrum of clinical symptoms from patients with mild symptoms to cardiogenic shock and death [35]. Although the incidence was low, the relative risk (RR) of developing symptomatic heart failure was 4.74 when bevacizumab was added to anticancer treatment regimens, compared with the addition of placebo [36]. A meta-analysis of studies including a total of 10,647 patients treated with TKIs for various cancers (including pazopanib, sorafenib and sunitinib) showed an overall incidence of asymptomatic LVSD of 2.4%, with 1.2% developing symptomatic heart failure [37]. A larger meta-analysis of 28,538 patients showed similar results. TKIs were associated with the highest RR for “high-grade” cardiotoxicity of 5.62, compared with placebo-treated patients. Of note, ramucirumab and aflibercept were also among the drugs with the highest relative risk of “high-grade” cardiotoxicity—5.0 and 4.1, respectively [38]. In a randomized trial of 1110 patients comparing pazopanib in combination with sunitinib for the treatment of renal cell carcinoma, the incidence of heart failure was 1%, while 9% of patients experienced a ≥15% reduction in LVEF [39]. The incidence of myocardial ischemia in patients treated with anti-VEGF antibodies is relatively low. In a meta-analysis of 4617 patients, bevacizumab-treated patients had a significantly increased risk of coronary heart disease (defined as myocardial infarction, unstable angina, coronary revascularization, coronary artery disease, arrhythmias, sudden death or cardiovascular death), compared with control (RR: 2.49). However, the overall incidence of coronary heart disease was only 1.0% [40]. In another meta-analysis of 72 clinical trials and 38,078 patients treated with VEGFI, 4136 cases of myocardial infarction were reported. VEGFI had a significantly increased relative risk of myocardial infarction (RR: 3.54) compared to the control group, although the absolute risk was only 0.8% [41].

Cardiac arrhythmias and prolongation of the QTc interval may also occur during anti-VEGF therapy, but the frequency and degree of increased risk of cardiac disorders associated with such therapy have not yet been specified. The TKIs most frequently associated with QTc prolongation are sorafenib and sunitinib. There are no reports of QTc prolongation with bevacizumab in clinical trials [26], and a study of 87 cancer patients treated with aflibercept, which has a higher affinity for binding VEGF, showed a mean increase in the QTc interval of only 8.4 ms [42]. Atrial fibrillation (AF) was most commonly reported following sorafenib administration. The incidence of AF was 5.1% when used in combination with 5-fluorouracil in a study of 39 patients with advanced hepatocellular carcinoma [43].

## 4. Mechanism of Cardiotoxicity Associated with the Use of Anti-VEGF Therapy

Cardiac muscle cells express all VEGF isoforms and VEGF receptor subtypes. This axis is stimulated by hypoxia and ischemia, as well as stretching of the myocardium. Cardiomyocytes release VEGF upon exposure to various stressors, which then act on vascular endothelial cells in a paracrine manner. Vascular endothelial cells also have the ability to release VEGF [10]. VEGF will contribute to cardiomyocyte turnover by activating mitogen-activated protein kinase (MAPK) pathways, which play a key regulatory role in cell proliferation in cardiomyocytes. The mitogenic effect of VEGF on endothelial cells is mediated by VEGFR-2 and activation of extracellular signal-regulated kinase (ERK1/2). The consequence of VEGFR-2 phosphorylation is the stimulation of the RAS/RAF/MAPK/ERK pathway in endothelial cells. In addition to its effects on VEGFR, sorafenib is a potent RAF inhibitor. VEGF also plays a role in cell migration, including endothelial cells. Cell migration is induced, among others, by the PI3K/Akt and MAPK pathways [26].

The role of AMPK (5′AMP-activated protein kinase), which is crucial for the energy homeostasis of cardiomyocytes, is also important. A decrease in ATP levels leads to the activation of AMPK and inhibition of protein and fatty acid synthesis. VEGF inhibitors can reduce the level of AMPK, which reduces its activation in response to ATP deficiency and, at the same time, affects the activation of mTOR [26].

Coronary vasoconstriction is regulated by the concentration of nitric oxide (NO). VEGF, by influencing VEGFR-2, causes its endothelial release. As a result of the use of VEGF inhibitors, the bioavailability of NO is reduced, which results in vasoconstriction, and is potentially the most important factor responsible for the occurrence of hypertension. Endothelin-1 (ET-1), responsible for vasoconstriction, may also play a role in this process. It has been shown that NO suppression and an increase in ET-1 concentration occurred in patients treated with regorafenib [26].

Moreover, NO, like prostacyclin (PGI2), has antiplatelet effects. Therefore, VEGFI-induced suppression of endothelial NO and PGI2 will result in increased coagulation. This increases the risk of arterial thrombosis and venous thromboembolism [26]. It has been shown that simultaneous administration of heparin with bevacizumab increases the deposition of bevacizumab-VEGF immune complexes on platelets, which leads to their activation [26].

Decreased production of nitric oxide and the resulting vasoconstriction may also contribute to the developing endothelial dysfunction, which promotes inflammation, atherosclerosis and platelet reactivity. Coronary vasoconstriction superimposed on structural changes may lead to a significant reduction in perfusion pressure and myocardial ischemia and failure [10].

One of the potential mechanisms of VEGFI-induced cardiac arrhythmias may be the inhibition of the PI3K/Akt pathway in cardiomyocytes. Binding of VEGF to VEGFR-2 on endothelial cells is associated with increased cell survival and migration through activation of the PI3K/Akt pathway. Inhibition of VEGF-induced signaling leads to a decrease in PI3K activity, among others, in the atrial appendage, leading to the occurrence of AF, which was proven in mouse models [26].

## 5. Cardiotoxicity Associated with the Use of Anti-PD-1/PD-L1 Therapy

The most frequently diagnosed cardiovascular irAE (immune-related adverse event) is myocarditis (79% of all cases), but arrhythmias may also occur, including atrial fibrillation (30%), conduction disturbances (17%) or ventricular arrhythmias (27%), pericardial diseases, vasculitis or takotsubo-like cardiomyopathy (14%) [12]. The incidence of myocarditis induced by anti-PD-1/PD-L1 therapy ranges from 0.09 to 2% but is associated with high mortality—up to half of cases. The onset of myocarditis associated with immunotherapy most often occurs at an early stage of treatment, with up to 80% occurring within the first 3 months after the initiation of therapy, i.e., most often after 2–3 treatment cycles [12]. Cardiac complications mostly lead to the termination of anti-PD-1/PD-L1 treatment.

Activated immune cells can attack not only myocardial but also pericardial antigens, which may lead to pericarditis with pericardial effusion or even cardiac tamponade. Pericarditis is a less common adverse event compared to myocarditis, with an incidence of up to 0.3%. Another adverse effect is vasculitis (with incidence estimated at approximately 0.26% [44]), most often polymyalgia rheumatica and temporal arteritis. The mortality rate for these complications is lower compared to other cardiovascular irAEs (6.1%), and visual disturbances were reported in 27.8% of cases of temporal arteritis [12].

Acute coronary syndrome associated with immunotherapy has an incidence of approximately 1%. A single-center study of 3326 patients undergoing immunotherapy showed a 7% incidence of myocardial infarction and a similar incidence of stroke over a mean follow-up period of 16 months [45].

## 6. Mechanism of Cardiotoxicity Associated with the Use of Anti-PD-1/PD-L1 Therapy

Cardiac complications also occur in the case of immunotherapy using PD-1/PD-L1 inhibitors. The PD-1/PD-L1 pathway seems to be crucial for immune homeostasis within the myocardium and for protection of the heart against T lymphocytes. The mechanism of cardiotoxicity induced by PD-1/PD-L1 inhibitors is not fully understood. One of the causes is immunological dysregulation in the heart muscle caused by excessive activation of native T lymphocytes. Another cause may be cross-reactions between T lymphocytes and antigens present in cardiac muscle cells. Dysregulated immune cells can falsely label surface structures such as cardiolipin as antigens. As a result, normal cardiomyocytes expressing such an antigen may become a target of the immune system. The third possible mechanism is a systemic immune response triggering the release of cytokines [12,44].

## 7. Nephrotoxicity Associated with the Use of Anti-VEGF Therapy

The incidence of nephrotoxicity associated with antiangiogenic therapy is estimated at 10–20%; however, compared to other irAEs, renal complications during immunotherapy are relatively rare [46]. The most common adverse effects of anti-VEGF therapy are hypertension, proteinuria and thrombotic microangiopathy. Their consequence may be a decrease in the glomerular filtration rate up to the development of acute kidney damage. In some cases, therapy with anti-VEGF drugs may lead to gradual but irreversible changes in renal function, up to end-stage renal disease [11]. The incidence of renal-related hypertension, depending on the source, ranges from 23 to 41% for bevacizumab, approximately 17–55% for sorafenib, 22–60% for sunitinib therapy and 40–52% for pazopanib [46]. The incidence of hypertension during the use of bevacizumab is dose dependent and rises with its increase [47]. In a randomized phase II trial in patients with advanced renal cell carcinoma treated with bevacizumab at a dose of 3 mg/kg, the incidence of hypertension was only 3%, compared to 36% in patients treated with 10 mg/kg [48]. The incidence of hypertension also increases with the simultaneous use of two antiangiogenic drugs. The combinations of bevacizumab and sunitinib and bevacizumab and sorafenib tested in clinical trials in advanced solid tumors, including mRCC, resulted in rates of 92% and 67%, respectively. Moreover, patients treated for kidney cancer have higher rates of hypertension than patients treated for other cancers, which is probably related to the history of nephrectomy [47].

In addition to hypertension, a common adverse effect associated with antiangiogenic agents is proteinuria. The incidence of all grades of proteinuria is approximately 10–20%. Most cases of proteinuria are asymptomatic, with proteinuria in the nephrotic range (>3 g/day) occurring in 1–5% of patients, depending on the duration of exposure to anti-VEGF therapy. Nephrotic proteinuria occurs with a higher frequency in patients with renal cell carcinoma (up to 7–8%) [46].

Bevacizumab-related proteinuria is dose dependent and occurs in 41–63% of patients [49]. Despite the relatively high incidence, most cases are asymptomatic and not severe, and proteinuria in the nephrotic range (>3.5 g/day) is reported in approximately 6.5% [48]. The incidence of proteinuria appears to be lower following the use of TKIs, compared to bevacizumab, and it is mostly without clinical significance; thus, no surveillance is re-quired in clinical practice [46]. The incidence of proteinuria is approximately 10% for sorafenib, 10–65% for sunitinib and 13.5–18% for pazopanib [46]. In one study, significant proteinuria (mean 3.8 g/day) occurred in 2.8% of 298 patients treated with sorafenib or sunitinib after a median treatment duration of 6 months [50,51]. With cabozatinib, proteinuria should be monitored and the drug should be stopped if grade 2 proteinuria occurs. The drug can be restarted at grade 1 proteinuria but at a reduced dose. Failure to monitor proteinuria may result in nephrotic syndrome [50].

Patients receiving concomitant intravenous bisphosphonates and/or nonsteroidal analgesics during anti-VEGF treatment have an increased risk of development or exacerbation of proteinuria [47].

In addition to the nephrotoxic effects of antiangiogenic agents, some drugs may cause damage to various tubular transporters. The most common electrolyte disorders include hypophosphatemia, hyponatremia, hypocalcemia and hypomagnesemia. Hypophosphatemia occurs in 16–85% of patients treated with sorafenib and 34% of patients treated with pazopanib. It is also observed during therapy with bevacizumab and sunitinib. Hyponatremia is observed in 39% of patients treated with sorafenib, 22–60% of patients treated with sunitinib and 31% of patients treated with pazopanib, and it may also occur during bevacizumab therapy [46].

## 8. Mechanism of Nephrotoxicity Associated with the Use of Anti-VEGF Therapy

VEGF plays a role in maintaining the integrity of the glomerulus. In the glomerulus, VEGF is expressed and secreted by podocytes located on the urinary side of the glomerular basement membrane. VEGFRs are expressed on the surface of both endothelial cells and podocytes. Several mechanisms have been suggested as the mechanism of renal-related hypertension in response to anti-VEGF therapy. The first is an increase in vascular tone caused by inhibition of vasodilation and an increase in vascular resistance in response to a decrease in NO concentration, similar to what happens in the heart vessels. Moreover, NO is involved in tubuloglomerular coupling, pressure natriuresis and sodium balance. Consequently, inhibition of NO signaling may lead to the development of hypertension through sodium retention. Another mechanism involved is reduction in fractional renal sodium excretion, which is reduced in tubular segments by antiangiogenic treatment, thereby contributing to volume-dependent hypertension. Tubular sodium reabsorption occurs through the epithelial sodium channel and the sodium chloride cotransporter in the distal convoluted tubule, communicating tubule and collecting duct. Epithelial cells of the renal distal tubules and collecting ducts express VEGFR2. A study conducted in mouse models showed that the use of sunitinib led to a significant reduction in diuresis and natriuresis, indicating that sunitinib stimulates sodium reabsorption in the kidneys.

ET-1 may also play a role in the development of hypertension. Inhibition of the VEGF pathway by antiangiogenic therapies induces ET-1 production in a dose-dependent manner. This causes hypertension and kidney dysfunction, in a mechanism similar to that of preeclampsia. Another mechanism of endothelial dysfunction is microcirculatory dilution, which may be induced by anti-VEGF therapy. Patients with chronic kidney disease are at increased risk of developing hypertension after anti-VEGF treatment [45,46].

VEGF is crucial for maintaining proper glomerular function. The interaction between VEGF produced by podocytes and VEGFR-2 on glomerular endothelial cells is essential to maintain glomerular membrane integrity. Reducing VEGF concentration may lead to decreased nephrin expression, which, in turn, leads to podocyte damage and proteinuria [51]. Inhibition of VEGF signaling, and thus inhibition of the mTOR signaling pathway through PI3K/Akt, may lead to disruption of autophagy, ultimately resulting in cell death and especially loss of podocytes. Autophagy is a metabolic process that helps cells maintain homeostasis by removing senescent or damaged organelles. Antiangiogenic agents reduce tumor microvascular density and increase tumor hypoxia, thus increasing tumor cell autophagy activation to maintain cell survival and normal metabolism. Increased autophagy is believed to play a cytoprotective role in most cases [46].

## 9. Nephrotoxicity Associated with the Use of Anti-PD-1/PD-L1 Therapy

Clinically, renal toxicity of anti-PD-1/PD-L1 therapy may manifest as acute kidney injury (AKI), proteinuria and dyselectrolytemia. The possible types of kidney damage that are most frequently described in the literature are acute tubulointerstitial nephritis, lupus-like glomerulonephritis, minimal change disease, membranous nephritis, focal segmental glomerulosclerosis and thrombotic microangiopathy [52].

The most frequently observed manifestation of nephrotoxicity caused by anti-PD1/PD-L1 therapy is the deterioration of kidney function, up to acute kidney damage. In one analysis, which included 3695 patients, the overall incidence of AKI in response to immunotherapy, including the use of PD1/PD-L1 inhibitors, was 3% [53]. The incidence of severe AKI, defined as a more than three-fold increase in serum creatinine (SCr) above baseline values, an increase in SCr to 4.0 mg/dl or the need for renal replacement therapy (dialysis), was 0.6% [53].

## 10. Mechanism of Nephrotoxicity Associated with the Use of Anti-PD-1/PD-L1 Therapy

The exact mechanisms of AKI associated with immunotherapy are poorly understood. As in the case of cardiotoxicity, activated T lymphocytes may play a role. The exact antigen in the case of kidneys has not yet been determined, but it is most likely present on renal tubular cells. Risk factors increasing the likelihood of AKI occurrence during anti-PD-1/PD-L1 therapy include simultaneous use of proton pump inhibitors (OR 2.38; 95%CI: 1.57–3.62), combined treatment with anti-CTLA-4 drugs (OR 2.71; 95%CI: 1.62–4.53) and lower baseline eGFR (OR 1.99; 95%CI: 1.43–2.76). The mainstay of treatment for immunotherapy-induced AKI is glucocorticosteroids. In one study, 103 of 119 patients (87%) treated with steroids experienced complete or partial renal regeneration [54].

## 11. Combination Treatment with Anti-PD-1/PD-L1 and Anti-VEGF Drugs

The rationale for combining anti-PD-1/PD-L1 drugs with anti-VEGF agents originates from the fact that VEGF impairs the function of anti-cancer T cells; hence, VEGF inhibition boosts T cells function and blocks checkpoints, which may significantly enhance antitumor therapy by conferring synergistic effects [55,56]. It has been proven that the combination of anti-PD-1/PD-L1 drugs with anti-VEGF drugs significantly improves clinical outcomes compared with monotherapy. Consequently, it has been approved and is now the standard of treatment, for instance, in the case of RCC (combination of pembrolizumab plus axitinib [57], avelumab plus axitinib [58] and nivolumab plus cabozantinib [59]), NSCLC (atezolizumab, bevacizumab and chemotherapy [60]) and HCC (combination of atezolizumab and bevacizumab [61]).

However, combining these two groups of drugs may elevate the incidence of complications, probably due to the two therapies mutually potentiating their cardiotoxicity [26]. The use of combination therapy was associated with an almost 5-fold increase in the risk of myocarditis. Additionally, combination therapy heightens the severity and mortality of associated myocarditis (mortality at 65.6% in combination therapy vs. 44.4% in monotherapy) [12]. The incidence of AKI among patients receiving combination therapy was higher and amounted to 5% [53].

The most common grade 3 or higher adverse events were hypertension (9.3%) and hyponatremia (3.6%). Deaths which occurred during the combination treatment were most often due to hemorrhage (18.8%), myocarditis (18.8%) and pneumonitis (12.5%) [61]. Interestingly, patients receiving anti-PD-1/PD-L1 therapy plus chemotherapy were at a significantly higher risk of treatment-related adverse events than patients receiving anti-PD-1/PD-L1 therapy plus targeted therapy. What is more, the overall incidences of adverse events were not significantly different between different cancer types [62].

Dual PD-L1/PD-1/VEGF inhibitors are already under investigation. Research conducted so far has shown that such agents may be effective and cause less toxicity compared to treatment with two monospecific drugs [63,64].

## 12. Single Nucleotide Polymorphisms and Adverse Events of Targeted Therapies

The severity of adverse effects of treatment with both TKI inhibitors and anti-VEGF drugs is thought to be related to genetic polymorphisms found in patients. The relationship between such polymorphisms and cardiac toxicity has been demonstrated in the case of treatment with anthracyclines or anty-HER2 drugs. For example, the shortening of the titin gene increases the risk of atrial fibrillation or cardiac failure in patients receiving anthracyclines or trastuzumab. Many studies have investigated the association between single nucleotide polymorphisms and excess risk of cardiovascular complications in patients undergoing treatment with anti-angiogenic therapies and immune checkpoint inhibitors [65,66].

In the case of bevacizumab, the increased risk of stage 3 and 4 hypertension was associated with the existence of single nucleotide polymorphisms (SNPs) in the WNK1, KLKB1 and GRK4 genes (OR = 6.45; *p* = 0.005; 95%CI, 1.86–22.39) [65]. A genome-wide meta-analysis of 1037 patients receiving bevacizumab found that the presence of SNPs in KCNAB1, TRIO and DNAH5 was related to an increased risk of hypertension and proteinuria. The rs6770663 and rs339947 variants were more than twice as frequent in patients with hypertension and proteinuria. The rs6770663 (A>G) variant in KCNAB1 increased the risk of hypertension (*p* = 4.16 × 10^−6^). However, the rs339947 (C>A) variant between TRIO and DNAH5 *p* = 1.59 × 10^−7^ was associated with proteinuria [56]. The presence of SNPs in EGF rs4444903 A>G, ELGN3 rs1680695 T>G may also significantly increase the risk of bevacizumab-induced hypertension (*p* = 0.0025, *p* = 0.012). Other polymorphisms increasing the risk of hypertension are associated with KDR, rs1870377 T>A and rs2305949 C>T [67].

The severity of adverse events during tyrosine kinase inhibitor (TKI) treatment is thought to be related to the occurrence of different haplotypes in patients. [68,69]. Sunitinib toxicity may be enhanced by the presence of VEGFA, endothelium-derived nitric oxide synthase (eNOS) and VEGFR3 polymorphisms [57]. Patients with the A allele of CYP3A4 (cytochrome P4503A4) rs4646437 had a more frequent occurrence of sunitinib-induced hypertension than wild-type carriers [59]. With axitinib, the presence of the rs2305948 C>T variant in VEGFR-2 increased the risk of elevated blood pressure [60]. The ABCB1 rs1045642 CT + TT (ABCB1—ATP binding cassette subfamily B member) genotype is related to an increasing risk of high blood pressure in patients undergoing treatment with sorafenib (*p* = 0.037) [70].

Similarly, in the case of ICL, the risk of adverse effects may be associated with the presence of SNPs in certain genes. Many polymorphisms have been identified in genome-wide association studies (GWAS) that have an impact on the immune response in the context of ICI drug therapy and its adverse effects [71]. Groha et al. found three variants that increased the incidence of severe adverse events (AEs) in a genome-wide association study of ICI-treated patients [72]. These variants were located near Il7 (rs16906115), IL22RA1 (rs75824728) and on 2p15 (rs113861051) [71]. These findings were confirmed in patients enrolled in the PD-L1 inhibitor atezolizumab trial. The relationships between the occurrence of single nucleotide polymorphisms (SNPs) and adverse effects of nivolumab treatment were also analyzed. Despite the detection of a large number of SNPs, no such association was found. Among the 90 polymorphisms analyzed, the strongest association was found with rs469490 [73]. Research into the relationship between SNPs and AEs is still ongoing and requires a large group of patients due to the diversity of the occurrence of changes.

## 13. Conclusions and Future Direction

Oncological therapy using anti-VEGF drugs and immunotherapy using anti-PD-1/PD-L1 drugs has changed the face of treatment of many cancers, significantly improving the achieved results, with relatively good tolerance. Most adverse events disappear after discontinuing the drug and, in most cases, leave no lasting organ damage. The decision whether to resume treatment after the occurrence of cardiovascular complications remains a problem. In 60–80% of cases, these complications are reversible after discontinuation of therapy with VSP inhibitors, unlike in the case of cardiotoxicity caused by the use of anthracyclines [28,29,30,31,32]. Cardiac complications may be distant in time and are still worth attention, especially for practitioners, due to their clinical consequences. The same applies to renal complications. In most patients, protein urine and high blood pressure disappear or improve significantly when anti-VEGF therapy is stopped [45,46,47,48,49]. In the vast majority of cases, complications related to immunotherapy can be controlled with the use of glucocorticosteroids [52].

The decision to resume treatment after controlling adverse effects related to, among others, cardiotoxicity and nephrotoxicity should be made after analyzing the risks and benefits it brings. Patients who have exhausted other therapeutic options constitute a special group. Especially in their case, the benefits of resuming treatment far outweigh the risk of recurrence of adverse effects.

The role of patient monitoring is invaluable and, in particular, periodic assessment of organ functions, which allows for the faster detection of abnormalities and their prevention. Searching for methods which could identify patients at increased risk of experiencing the discussed adverse events, or developing drugs which could be used to control such events without the need of therapy discontinuation, is something to focus on in upcoming years, along with further advancement of immunotherapy and targeted therapy.

## Figures and Tables

**Table 1:** Examples of anti-VEGF and anti-PD-1 drugs, their targets, possible toxicity and use in cancer therapy.

Angiogenesis Inhibitors					
Drug	Type of Agent	Target	Approval by FDA in Cancer Treatment	Main Adverse Effects	References
Bevacizumab	Humanized monoclonal antibody	VEGF-A	Colorectal cancer, glioblastoma, cervical cancer, NSCLC, ovarian cancer, RCC	hypertension, bleeding, thrombo-embolism, gastro-intestinal perforations, proteinuria	[6,7,8]
Aflibercept	Soluble decoy receptor	VEGF-A, VEGF-B, PIGF	Colorectal cancer	Bleeding, perforation of the digestive tract, hypertension, thrombo-embolism formation of a fistula, heart failure, decreased ejection fraction	[6,7]
Ramucirumab	Human monoclonal antibody	VEGFR-2	Gastric cancer, NSCLC, colorectal cancer	hypertension, diarrhea, headache, hyponatremia, anaemia, intestinal obstruction	[6]
Sunitinib	Small-molecule TKI	VEGFR-1, VEGFR-2, PDGFR, KIT, FLT3	Gastrointestinal stromal tumor, pancreatic neuroendocrine tumor, RCC	hypertension, nausea, vomiting, diarrhea, fatigue, lymphopenia, neutropenia	[6,7,8]
Sorafenib	Small-molecule TKI	VEGFR-1, VEGFR-2, Raf, PDGFR, KIT, RET	Hepatocellular carcinoma, RCC, differentiated thyroid cancer	hypertension, nausea, vomiting, diarrhea, fatigue, anorexia, hand-food syndrome and rash	[6,7,8]
Pazopanib	Small-molecule TKI	VEGFR-1, VEGFR-2, VEGFR-3	RCC, soft tissue sarcoma	hypertension, nausea, vomiting, diarrhea	[6,7]
Axitinib	Small-molecule TKI	VEGFR-1, VEGFR-2, VEGFR-3	RCC	hypertension, nausea, vomiting, diarrhea, fatigue, stomatitis	[7,8]
Vantedanib	Small-molecule TKI	VEGFR-2, EGFR, FGFR1, RET	Medullary thyroid cancer	hypertension, nausea, vomiting, diarrhea, fatigue, weight loss	[7,8]
Immune checkpoint inhibitors					
Nivolumab	Human monoclonal antibody	PD-1	melanoma, NSCLC, pleural mesothelioma, renal cell carcinoma, HNSCC, urothelial carcinoma, colorectal cancer, HCC, esophageal cancer, gastroesophageal junction cancer, gastric cancer	pruritus, fatigue, loss of appetite, immune related-adverse events (irAE)—dermatitis, hypophysitis, colitis and hepatitis	[9]
Pembrolizumab	Humanized monoclonal antibody	PD-1	melanoma, NSCLC, HNSCC, urothelial carcinoma, gastric cancer, colorectal cancer, esophageal cancer, cervical cancer, HCC, RCC, endometrial carcinoma, triple-negative breast cancer, cutaneous squamous cell carcinoma, Merkel cell carcinoma		[9]
Cemiplimab	Human monoclonal antibody	PD-1	NSCLC, squamous cell skin cancer		[9]
Atezolizumab	Humanized monoclonal antibody	PD-L1	NSCLC, SCLC, HCC, melanoma, alveolar soft part sarcoma		[9]
Avelumab	Humanized monoclonal antibody	PD-L1	Merkel Cell carcinoma, urothelial carcinoma, RCC		[9]
Durvalumab	Humanized monoclonal antibody	PD-L1	NSCLC, SCLC, biliary tract cancer, HCC		[9]

Abbreviations: VEGFR—vascular endothelial growth factor receptor, VEGF—vascular endothelial growth factor, PD-1—programmed cell death protein 1 receptor, PD-L1—programmed cell death protein 1 ligand, FGFR—fibroblast growth factor receptor, PDGFR—platelet-derived growth factor receptor, PIGF—placental growth factor, KIT—receptor tyrosine kinase, FLT3—Fms-like tyrosine kinase 3, RET—ret proto-oncogene, HCC—hepatocellular carcinoma, HNSCC—squamous cell carcinoma of the head and neck, NSCLC—non-small cell lung cancer, SCLC—small cell lung cancer, RCC—renal cell carcinoma.
